# Review of "Stochastic Modelling for Systems Biology" by Darren Wilkinson

**DOI:** 10.1186/1475-925X-5-64

**Published:** 2006-12-13

**Authors:** Eric Bullinger

**Affiliations:** 1 Hamilton Institute, National University of Ireland, Maynooth, Co. Kildare, Ireland

## Abstract

"Stochastic Modelling for Systems Biology" by Darren Wilkinson introduces the peculiarities of stochastic modelling in biology. This book is particularly suited to as a textbook or for self-study, and for readers with a theoretical background.

Gene expression as well as reactions involving low protein concentrations are intrinsically stochastic, see e.g. [1]. In biology, this is called intrinsic noise, while extrinsic noise refers to cell-to-cell variation due for example to different expression levels of proteins. This book gives an introduction to stochastic modelling with a particular focus on chemical reactions and intrinsic noise. Whether stochastic modelling is "the best way" as the author claims certainly depends on the objective of the modelling as well as on the availability of data which lead to a specific choice of modelling framework, see for example [2] for an overview of different frameworks, one of which is stochastic kinetic modelling. Chapter 1 motivates stochastic modelling and describes how to obtain models (lists of reactions) of exemplary biochemical processes. Chapter 2 shows how to transform these lists of reactions into equivalent petri nets as well as how to describe them using the Systems Biology Markup Language (SBML) [3]. SBML is a format for representing models of biochemical reaction networks supported by over 100 software systems and thus a de-facto standard. Chapter 3 presents distributions such as the uniform, Poisson or gamma distributions. Of particular importance in stochastic modelling is the exponential distribution, which is often used to model the times between consecutive reaction. The book lists several statistic results with proofs of why such a distribution makes sense. A key step in numerical solvers of stochastic models is the calculation of exponentially distributed numbers. This is discussed in Chapter 4 in combination with an introduction to R, a language and environment for statistical computing and graphics [4]. Chapter 5 discusses discrete and continuous-time Markov processes. Chapter 6 finally presents numerical solutions of biochemical reaction models, from ordinary differential equation models over the Gillespie algorithms and stochastic Petri nets to the master equation. The following chapter proposes three small and a larger model (11 species, 16 reactions) of the lac-operon. Chapter 8 discusses extensions/modification of the Gillespie algorithms allowing faster simulations in an exact or approximate way. The final two chapters present model identification for stochastic models, an area of cutting edge research.

Most textbooks only cover deterministic models and this book is the first to offer a comprehensive introduction into the theory of and around the Gillespie algorithm. A more compact discussion is given by Gillespie and Petzold in [5], Chapter 16. The book under review is however designed for and well suited as a book for an in-depth introduction into stochastic chemical simulation, both for self-study or as a course text, but less as a reference book. The examples, whose code as well as complementary links can be found on the author's web page [6] certainly help in illustrating the theory and concepts. The ideal target audience are probably advanced graduate students with an engineering, mathematical or similarly theoretical background. For biologists, the book style is probably too abstract.
